# Factors associated with suffering from dying in patients with cancer: a cross-sectional analytical study among bereaved caregivers

**DOI:** 10.1186/s12904-023-01148-x

**Published:** 2023-04-21

**Authors:** Angélica Arango-Gutiérrez, Socorro Moreno, Martín Rondón, Lucía I Arroyo, Liliana Ardila, Fabián Alexander Leal Arenas, José A Calvache, Esther de Vries

**Affiliations:** 1 MSc programme Clinical Epidemiology, Pontificia Universidad Javeriana, Bogotá, Colombia; 2 Department of Clinical Epidemiology and Biostatistics, Pontificia Universidad Javeriana, Bogotá, Colombia; 3 Departamento de Fonoaudiología, Universidad de Cauca, Popayán, Colombia; 4 MSc programme Public Health, Pontificia Universidad Javeriana Cali, Cali, Colombia; 5 National Cancer Institute of Colombia, Bogotá, Colombia; 6 Javeriano Oncology Center, Hospital Universitario San Ignacio, Bogotá, Colombia; 7 Department of Anaesthesiology, Universidad del Cauca, Popayán, Colombia; 8 Department of Anaesthesiology, Erasmus MC University Medical Center Rotterdam, Dr. Molewaterplein 40, Rotterdam, 3015 GD The Netherlands

**Keywords:** Suffering, Neoplasms, Caregivers, Medical futility, Palliative care, End of life, Communication

## Abstract

**Background:**

In Colombia, cancer incidence is increasing, as is the demand for end-of-life care. Understanding how patients who die from cancer experience this phase will allow the identification of factors associated with greater suffering and actions to improve end-of-life care. We aimed to explore associations between the level of suffering of patients who died from cancer and were cared for in three Colombian hospitals with patient, tumor, treatment, and care characteristics and provided information.

**Methods:**

Data on the last week of life and level of suffering were collected through proxies: Bereaved caregivers of patients who died from cancer in three participating Colombian hospitals. Bereaved caregivers participated in a phone interview and answered a series of questions regarding the last week of the patient’s life. An ordinal logistic regression model explored the relationship between the level of suffering reported by bereaved caregivers with the patient’s demographic and clinical characteristics, the bereaved caregivers, and the care received. Multivariate analyses were adjusted for place of death, treatments to prolong of life, prolongation of life during the dying process, suffering due to prolongation of life, type of cancer, age, if patient had partner, rural/urban residence of patient, importance of religion for the caregiver, caregivers´ relationship with the patient, and co-living with the patient.

**Results:**

A total of 174 interviews were included. Median age of the deceased patients was 64 years (IQR 52–72 years), and 93 patients were women (53.4%). Most caregivers had rated the level of suffering of their relative as “moderately to extremely” (n = 139, 80%). In multivariate analyses, factors associated with a higher level of suffering were: unclear information about the treatment and the process before death Odds Ratio (OR) 2.26 (90% CI 1.21–4.19), outpatient palliative care versus home care OR 3.05 (90% CI 1.05–8.88), procedures inconsistent with the patient’s wishes OR 2.92 (90% CI 1.28–6.70), and a younger age (18–44 years) at death versus the oldest age group (75–93 years) OR 3.80 (90% CI 1.33–10.84, p = 0.04).

**Conclusion:**

End-of-life care for cancer patients should be aligned as much as possible with patients´ wishes, needs, and capacities. A better dialogue between doctors, family members, and patients is necessary to achieve this.

**Supplementary Information:**

The online version contains supplementary material available at 10.1186/s12904-023-01148-x.

## Background

The demographic transition due to the aging of the population has brought changes in the leading causes of death, including the increase in mortality from cancer. Worldwide, it is estimated that by 2020, there were 9.9 million deaths from cancer, excluding non-melanoma skin cancer [1].

Cancer often has extended end-of-life processes. Continuous technological advances have allowed the development of treatment strategies that prolong survival, including in “incurable” settings, when death is inevitable but can be postponed [2, 3]. Whereas this postponement of death may be desirable in some situations, it may be undesirable if it prolongs suffering or results from non-acceptance of imminent death, sometimes reflecting failures in the information process regarding the prognosis with the treating physician [4], or denial of a natural process of life by patients and family members.

Palliative care is care without curative intentions, focused on symptom relief, and can be offered to patients at any stage of the disease, helping to improve quality of life and coping with grief [5]. Palliative care was born in the mid-20th century to achieve adequate control of all kinds of disease symptoms and an accompaniment to relatives and patients in the process of coping with the end-of-life and death—aiming to improve both quality of life and quality of death. Despite the benefits that palliative care offers, referral is often in late stages [6] and many cancer patients receive treatments with curative intent even at the end-of-life stage without referral to palliative care – both situations have been described to be associated with unnecessary suffering [6].

Suffering is a multidimensional concept that is not just influenced by physical manifestations, but also by other factors such as disease status, social and physical environment, received care and the loss of roles (social roles, identification within groups, relations with self and body, etc. [7–9]. The probability of receiving palliative care differs by type of cancer: patients diagnosed with hematological cancer are less likely to receive palliative care than those with a solid cancer and more likely to receive intensive treatment at the end of life and to die in hospital [10–12].

Colombia has recently implemented legislation to guarantee access to palliative care but access is “universal” only on paper [13], even though its healthcare system covers to 99.4% of its population. This coverage is through two major insurance schemes: The subsidized scheme (47.2%), for people without payment capacity, and the contributory scheme (48.5%), which is financed by contributions from the labor force (employees and employers), there is also an exceptional regime (4.2%) for workers from the public force and some institutions [14, 15].The offer of medical services between regions of Colombia varies substantially, with absence of many services in many regions, implying patients from more remote areas move to major cities to receive treatment and care. This situation translates to differences in the opportunity to access palliative care [13], even among those affiliated, and may lead to hospital deaths far from the patients´ homes or homes deaths with an absence of medical care [12].

Being able to decide on the preferred place of death is internationally considered an indicator of the quality of the end of life, with most patients usually stating a preference to die at home [16, 17]. In Colombia, of all patients who died from cancer between 2014 and 2017, 31.1% died at home, most of whom were patients with low educational level, living in rural areas and many without social security system affiliation; their place of death likely reflects difficulties in access to medical treatment rather than “a death at the preferred place”, although this aspect has not yet been explored in depth [12].

Another important factor during cancer patient care is proper patient-doctor communication. However, many Colombian health care professionals indicate not to feel prepared to communicate with the patient and family, especially during the first interactions [18] or when they must inform the patient that there are no curative options, and that patients will probably die due to their cancer.

We know very little regarding the context and quality of dying of cancer patients in Colombia. The aforementioned factors, added to the characteristics of our population, may affect the level of suffering experienced by cancer patients at the end of their life. As a first approximation to these topics, our aim was to measure suffering at the end of life of patients who died of cancer and were attended for in three Colombian hospitals. We explored associations between this suffering and place of death, rural/urban residence of patient, type of cancer, clarity in the information received by the doctor, greater suffering due to prolongation of life, procedures inconsistent with the wishes, treatments to prolong of life, prolongation of life during the dying process and palliative care modality.

## Methods

### Study design

We conducted a cross-sectional study as part of a larger study: “Medical decisions at the end of life in cancer patients in Colombia” [19]. In this study, between 2019 and 2020, caregivers were contacted of deceased adult cancer patients who received care at one of the three participating hospitals (Hospital Universitario San Ignacio (HUSI) in Bogota, Instituto Nacional de Cancerología (INC) in Bogota, and Hospital Universitario San José (HUSJ) in Popayán). Caregivers who voluntarily agreed to participate by means of a recorded verbal consent were eligible for our research; those who did not complete the global measurement of suffering question were excluded from the analysis.

### Variables

In our study, we included as independent variables some questions from the Caregiver’s Evaluation of the QUality of End-of-Life care scale (CEQUEL) [20], and the Quality of Dying and Death scale (QODD) [21]. In addition, we included some questions that corresponded to the demographic and clinical characteristics of the patients (See additional file 1, supplementary Table 1) [19].

We measured suffering at the end of life using the following question: “From 1 to 7, to what extent do you think [patient’s name] suffered in dying?” [22]. This question performs a global measurement of suffering at the time of death of a patient as reported by the caregiver –and as a proxy to the construct of suffering proposed by Cassell [7].

### Data collection and management

Three trained health professionals conducted the interviews through telephone calls, they had a script to invite and explain the project to the caregiver. Data was collected digitally through the SurveyMonkey platform; no identifying data of neither deceased patient nor caregiver was recorded and each interview was given an alphanumeric identifier. The present study’s database was transferred to our analysis and guarded with an access code to restrict its use.

### Data analysis

The R software (version 4.0.2 (2020-06-22) [23]), was used for the analyses. To verify a potential influence of non-response bias, a Χ^2^ distribution homogeneity test was performed between identified patients. We were able to obtain a response from a family member or caregiver (responders) versus those who did not respond (non-responders). We compared the following variables that were extracted from the clinical history of deceased patients for both groups (responders and non-responders): sex, whether they had a partner or not, type of affiliation to the social security system, type of cancer, age, and center of care of a patient.

Frequency of responses given to the global measurement of suffering question was calculated after recategorizing the response options as follows: where 1&2 = suffered minimally, 3–5 = suffered moderately, 6&7 = suffered extremely. We established these thresholds for re-categorization, as they more accurately reflected the distribution of scores, avoiding floor-to-ceiling effects.

To evaluate associations between the outcome (level of suffering) and demographic characteristics, characteristics of the caregiver, and type of treatment, a bivariate analysis was initially performed with a chi-square test. If a variable presented an expected frequency of less than five in more than 20% of the cells, a Fisher exact test was performed.

We performed an ordinal logistic regression model to study the relationship between the level of suffering of patients who died from cancer with the factors of interest that had previously been reported in the literature: rural/urban residence of patient [12, 13], place of death [16, 17], type of cancer [10–12], clarity in the information received by the doctor [3, 18], greater suffering due to prolongation of life [2–4], procedures inconsistent with the wishes [3], treatments to prolong of life [6], prolongation of life during the dying process [2–4, 6], palliative care modality [6].

The reference categories of the ordinal logistic regression model were chosen according to the categories that we expected to have the least suffering. The estimates of the model were presented as ORs and their uncertainty by 90% CI. The alpha level of 0.1 and corresponding 90% confidence intervals were chosen to reduce the probability of type II errors, which we considered of importance in this exploratory study.

The following independent variables were considered *a priori* as potential confounding variables: the patient had a partner (with partner/single), care center (HUSI/HUSJ/INC), sex (man/woman), and age of the patient (18–44; 45–59; 60–74; 75–93), educational level achieved by the patient (no formal education; basic; university-higher education; unknown), patient’s health care affiliation scheme (contributory; subsidized) [13], the time between the patient’s death and the interview (in weeks), caregivers´ relationship with the patient (other; partner; father-mother-son-daughter; brother-sister), coliving with the patient (yes/no), the importance of religion for the caregiver (very important; important; not important), and gender of the caregiver (male; female). Variables on this list were included as confounding variable if excluding them had an effect of 20% or more in the parameter estimates of the factors of interest. Factors of interest were maintained in the final model regardless of their statistical significance.

The following interactions between some independent variables (factors of interest) and some covariates were proposed *a priori*: (a) as we expected the educational level might influence the perception of the clarity of information: interaction between clear information received by the medical staff and educational level of the patient; (b) A less close relationship between the caregiver and patient may have influenced the caregivers´ knowledge regarding whether or not procedures that were performed were inconsistent with patients´ wishes: interaction between caregivers´ relationship with the patient and report of procedures inconsistent with patients´ wishes; (c) as the type of contracting of health insurances with cancer centers may influence which modality of care is offered, we explored the interaction between care center and palliative care modality; and finally, (d) as a lack of clarity of information received by the doctors may lead to procedures inconsistent with patients´ wishes we explored the interaction between these variables.

We evaluated compliance with the assumption of proportionality of odds through the Wald test for each independent variable. A likelihood ratio test was performed to assess whether the fit of the reduced model is as good as that of the entire model.

The hypothesis contrast for each one of the estimators of the coefficients was made using the Wald test with α = 0.10, for the other tests α = 0.05 was used.

## Results

### Study population

**Fig. 1:**
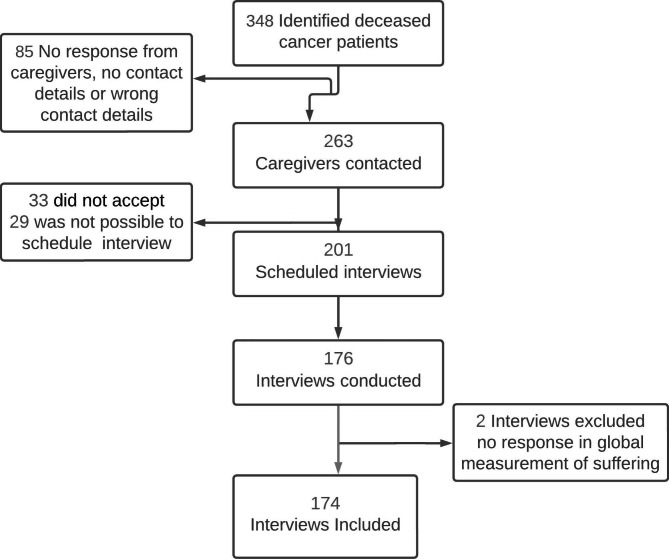
Identified deceased cancer patients and interviewed caregivers

We tried to contact all 348 caregivers of patients who died of cancer, identified in the main study [19], and managed to establish contact with 263 of them. In total, 176 interviews were conducted, and those who did not answer the global measurement of the level of suffering were excluded (n = 2) (See Fig. 1). We used the available data of the 174 caregivers who answered the level of suffering question.

The comparison between the baseline characteristics of the responders and the non-responders shows a comparable distribution of most measured characteristics between both groups, except for the living situation of the deceased patient: The group of responders presented a higher percentage of patients with a partner (n = 120, 69.0%) compared to non-responders (n = 77, 48.7%, p < 0.001), (see Table 1).

**Table 1:** Comparison of characteristics between patients with and without a response from caregivers and results of test of homogeneity

	Answered No (N = 174)	Answered Yes (N = 174)	Overall (N = 348)	p-value*
**Age**				0.477
18**–**44	35 (20.1%)	28 (16.1%)	63 (18.1%)	
45**–**59	35 (20.1%)	43 (24.7%)	78 (22.4%)	
60**–**74	62 (35.6%)	68 (39.1%)	130 (37.4%)	
75**–**93	42 (24.1%)	35 (20.1%)	77 (22.1%)	
**Sex**				0.914
Male	79 (45.4%)	81 (46.6%)	160 (46.0%)	
Female	95 (54.6%)	93 (53.4%)	188 (54.0%)	
**Patient had partner**				< 0.001
Yes	77 (48.7%)	120 (69.0%)	197 (59.3%)	
No	81 (51.3%)	54 (31.0%)	135 (40.7%)	
**Type of cancer**				0.723
Hematological	18 (11.1%)	17 (9.8%)	35 (10.4%)	
Solid	144 (88.9%)	157 (90.2%)	301 (89.6%)	
**Center of attention**				0.842
HUSI	77 (44.3%)	73 (42.0%)	150 (43.1%)	
HUSJ	17 (9.8%)	15 (8.6%)	32 (9.2%)	
INC	80 (46.0%)	86 (49.4%)	166 (47.7%)	
**Health affiliation scheme**				0.319
Contributory	103 (59.2%)	104 (59.8%)	207 (59.5%)	
Special/exceptional	3 (1.7%)	0 (0.0%)	3 (0.9%)	
Private	1 (0.6%)	0 (0.0%)	1 (0.3%)	
Subsidized	67 (38.5%)	70 (40.2%)	137 (39.4%)	

HUSI: Hospital Universitario San Ignacio, Bogotá; HUSJ: Hospital Universitario San José, Popayán; INC = Instituto Nacional de Cancerología, Bogotá

*Test for homogeneity

Demographic characteristics of the deceased patients for whom we obtained a response from the caregiver are described in Table 2. The median age of the deceased patients was 63.5 years (IQR 52–72 years), 103 patients (59.2%) were 60 years of age or older, 93 patients were women (53.4%), most (n = 157, 90.2%) had solid cancer, 104 patients (59.8%) were from the contributory regime, and 20 patients (11.5%) had no formal education.

Most of the patients with participating caregivers had children (n = 161, 92.5%), mostly adults (n = 129, 74.1%). The vast majority of patients lived accompanied by someone from their nuclear family (n = 164, 94.2%), and most patients lived in urban areas (n = 132, 75.9%) and had a partner (n = 120, 69.0%).

**Table 2:** Demographic variables of the deceased patients

Variables	Overall (N = 174)
Age*	
Median	63.5
Q1, Q3	52.0, 72.0
**Age**	
18–44	28 (16.1%)
45–59	43 (24.7%)
60–74	68 (39.1%)
75–93	35 (20.1%)
**Sex**	
Male	81 (46.6%)
Female	93 (53.4%)
**Patient had partner**	
Yes	120 (69.0%)
No	54 (31.0%)
**Type of cancer diagnosed**	
Solid	157 (90.2%)
Hematological	17 (9.8%)
**Health affiliation scheme**	
Contributory	104 (59.8%)
Subsidized	70 (40.2%)
**Educational level reached**	
No formal education	20 (11.5%)
Basic**	114 (65.5%)
University/higher education	38 (21.8%)
Unknown	2 (1.1%)
**Rural/urban residence of patient**	
Urban	132 (75.9%)
Municipal head	18 (10.3%)
Scattered rural	24 (13.8%)
**Offspring**	
Yes	161 (92.5%)
No	13 (7.5%)
**Children’s ages**	
Children aged ≥ 18	129 (74.1%)
Children < 18	30 (17.2%)
Does not know	2 (1.1%)
**Living condition**	
Other(s)	69 (39.7%)
Lives alone	10 (5.7%)
Lives with partner	28 (16.1%)
Lives with partner and children	55 (31.6%)
Lives with children	10 (5.7%)
Lives with parents	2 (1.1%)

*Normality test result: W = 0.97957, p = 0.01

**Primary, secondary, and technical education

The median number of weeks elapsed between the patient’s death and the interview with caregivers was 23.8 (IQR 22.0, 27.8). The interviewed caregivers were family members (n = 127, 73.0%) or partner (n = 33, 19.0%) of the patient, the vast majority were women (n = 132, 75.9%), and they were “very much involved” in the care for the patient (n = 161, 92.5%). Half of the caregivers lived with the patient (n = 88, 50.6%) and, for most, religion was essential (n = 165, 94.8%).

The participation of caregivers by center was similar with 48.7% (N = 73) from HUSI 51.8% (N = 86), from INC, and 46.9% (N = 15) from HUSJ. Most patients died in hospital (n = 148, 85.1%) and were accompanied at the moment of death (n = 159, 92.4%).

The distribution of the patient’s level of suffering at death according to their caregivers was: 1&2 = minimally (n = 35, 20.1%), 3–5 = moderately (n = 56, 32.2%) and 6&7 = extremely (n = 83, 47.7%).

Regarding medical care received during the last weeks of life, most patients (n = 154, 88.5%) received palliative care in its different modalities. A considerable number of caregivers (n = 60, 34.5%) stated that the patient’s life was prolonged while they were in the process of dying, and that having performed interventions to prolong the patient’s life had increased their suffering (n = 41, 23.6%). Of these 41 patients, 35 (85.4%) received palliative care.

The information provided by the doctors was qualified as “unclear” by 51 caregivers (29.3%); in addition, 25 (14.4%) of caregivers said that procedures inconsistent with the wishes of the patient were performed.

The bivariate analysis results showed no differences in patients’ and caregivers’ sociodemographic characteristics between the level of suffering. Regarding treatment received in the last week of life, results showed a relationship between the level of suffering and the clarity of the information received by the doctor (p = 0.038), (Tables 3 and 4).

**Table 3:** Bivariate analysis with demographic variables and caregiver characteristics

Variable	Minimally (N = 35)	Moderately (N = 56)	Extremely (N = 83)	p-value
Patient age*				0.157
18–44	2 (5.7%)	6 (10.7%)	20 (24.1%)	
45–59	10 (28.6%)	13 (23.2%)	20 (24.1%)	
60–74	17 (48.6%)	24 (42.9%)	27 (32.5%)	
75–93	6 (17.1%)	13 (23.2%)	16 (19.3%)	
Sex*				0.195
Male	21 (60.0%)	25 (44.6%)	35 (42.2%)	
Female	14 (40.0%)	31 (55.4%)	48 (57.8%)	
Type of cancer diagnosed**				0.841
Solid	31 (88.6%)	50 (89.3%)	76 (91.6%)	
Hematological	4 (11.4%)	6 (10.7%)	7 (8.4%)	
Patient health affiliation scheme*				0.320
Contributory	20 (57.1%)	38 (67.9%)	46 (55.4%)	
Subsidized	15 (42.9%)	18 (32.1%)	37 (44.6%)	
The educational level reached by the patient**				0.422
No formal education	6 (17.1%)	3 (5.4%)	11 (13.3%)	
Basic	22 (62.9%)	39 (69.6%)	53 (63.9%)	
University	6 (17.1%)	14 (25.0%)	18 (21.7%)	
Does not know	1 (2.9%)	0 (0.0%)	1 (1.2%)	
Rural/urban residence of patient**				0.827
Urban	27 (77.1%)	41 (73.2%)	64 (77.1%)	
Municipal head	2 (5.7%)	7 (12.5%)	9 (10.8%)	
Scattered rural	6 (17.1%)	8 (14.3%)	10 (12.0%)	
Offspring**				0.414
Yes	32 (91.4%)	54 (96.4%)	75 (90.4%)	
No	3 (8.6%)	2 (3.6%)	8 (9.6%)	
Children’s ages**				0.104
Does not have children	3 (8.6%)	2 (3.6%)	8 (9.6%)	
Children aged ≥ 18	30 (85.7%)	45 (80.4%)	54 (65.1%)	
Children < 18	2 (5.7%)	9 (16.1%)	19 (22.9%)	
Does not know	0 (0.0%)	0 (0.0%)	2 (2.4%)	
**Caregiver characteristics**				
Importance of religion**				0.449
Very important	24 (68.6%)	39 (69.6%)	52 (62.7%)	
Important	11 (31.4%)	15 (26.8%)	24 (28.9%)	
Not important	0 (0.0%)	2 (3.6%)	7 (8.4%)	
Caregivers´ relationship with the patient**				0.661
Other	2 (5.7%)	3 (5.4%)	9 (10.8%)	
Partner	7 (20.0%)	12 (21.4%)	14 (16.9%)	
Father/Mother/Son	19 (54.3%)	36 (64.3%)	48 (57.8%)	
Brother/sister	7 (20.0%)	5 (8.9%)	12 (14.5%)	
Coliving with the patient*				0.480
Yes	19 (54.3%)	31 (55.4%)	38 (45.8%)	
No	16 (45.7%)	25 (44.6%)	45 (54.2%)	
Sex*				0.144
Male	4 (11.4%)	15 (26.8%)	23 (27.7%)	
Female	31 (88.6%)	41 (73.2%)	60 (72.3%)	
**Weeks between the interview and the death of the patient**				0.616
Number	35	56	83	
Median	24.5	23.1	23.8	
Q1, Q3	21.7, 28.7	21.4, 27.5	22.2, 27.1	

*Pearson’s chi-squared test

**Fisher’s exact test

**Table 4:** Bivariate analysis with treatment received in the last week of life

Variable	Minimally (N = 35)	Moderately (N = 56)	Extremely (N = 83)	p-value
Place where the patient died*				0.829
Home	6 (17.1%)	9 (16.1%)	11 (13.3%)	
Hospital	29 (82.9%)	47 (83.9%)	72 (86.7%)	
Center of attention**				0.760
HUSI	15 (42.9%)	26 (46.4%)	32 (38.6%)	
HUSJ	4 (11.4%)	5 (8.9%)	6 (7.2%)	
INC	16 (45.7%)	25 (44.6%)	45 (54.2%)	
Treatments to prolong of life*				0.650
Yes	8 (22.9%)	9 (16.1%)	18 (21.7%)	
No	27 (77.1%)	47 (83.9%)	65 (78.3%)	
Prolongation of life during the dying process*				0.526
Yes	10 (28.6%)	18 (32.1%)	32 (38.6%)	
No	25 (71.4%)	38 (67.9%)	51 (61.4%)	
Procedures inconsistent with patient wishes*				0.141
Yes	2 (5.7%)	7 (12.5%)	16 (19.3%)	
No	33 (94.3%)	49 (87.5%)	67 (80.7%)	
Palliative care modality**				0.341
Domiciliary	5 (14.3%)	4 (7.1%)	7 (8.4%)	
Ambulatory	6 (17.1%)	22 (39.3%)	27 (32.5%)	
Hospitalized	19 (54.3%)	26 (46.4%)	38 (45.8%)	
Did not receive	5 (14.3%)	4 (7.1%)	11 (13.3%)	
Greater suffering due to prolongation of life*				0.291
Yes	5 (14.3%)	13 (23.2%)	23 (27.7%)	
No	30 (85.7%)	43 (76.8%)	60 (72.3%)	
Clarity in the information received by the doctor*				0.038
Yes	28 (80.0%)	44 (78.6%)	51 (61.4%)	
No	7 (20.0%)	12 (21.4%)	32 (38.6%)	

*Pearson’s chi-squared test

**Fisher’s exact test

The outcome variable level of suffering was initially recorded on a scale from 1 to 7, which was reduced to 3 categories. As the statical model complied with the proportionality assumption (see additional file 1, supplementary Table 2), the Odds Ratios (ORs) can be interpreted dichotomously, meaning that we can assume that the probability of minimally or moderately versus extremely equals the probability of minimally versus moderately or severely.

The factors associated with a higher level of suffering in the multivariate ordinal logistic regression model were: unclear versus clear information received by the medical staff (OR 2.26 (90%CI 1.21–4.19)), ambulatory versus domiciliary palliative care (OR 3.05 (90%CI 1.05–8.88)), and the patient having received procedures inconsistent with his/her wishes (OR 2.92 (90%CI 1.28–6.70)) (Table 5).

A younger age (18–44 years) at death was associated with a higher reported level of suffering compared to the oldest age group (75–93 years) (OR 3.80 (90% CI 1.33–10.84, p = 0.04)). Age confounded the relations between the level of suffering and the variables: “type of cancer”, “palliative care modality” and “prolongation of life during the dying process”. None of the proposed interactions was statistically significant.

**Table 5:** Results of the ordinal logistic regression model

Variables	OR^1^	90% CI		p-value
**Place where the patient died**				
Home (reference)				
Hospital	1.18	(0.50;	2.79)	0.74
**Clarity in the information received by the doctor**				
Yes (reference)				
No	2.26	(1.21;	4.19)	0.03*
**Palliative care modality**				
Domiciliary (reference)				
Ambulatory	3.05	(1.05;	8.88)	0.09*
Hospitalized	1.42	(0.47;	4.30)	0.60
Did not receive	1.56	(0.40;	6.08)	0.59
**Procedures inconsistent with the wishes**				
No (reference)				
Yes	2.92	(1.28;	6.70)	0.03*
**Treatments to prolong of life**				
No (reference)				
Yes	0.98	(0.46;	2.09)	0.97
**Prolongation of life during the dying process**				
No (reference)				
Yes	1.44	(0.75;	2.74)	0.36
**Greater suffering due to prolongation of life**				
No (reference)				
Yes	1.33	(0.62;	2.84)	0.53
**Type of cancer**				
Solid (reference)				
Hematological	0.52	(0.21;	1.31)	0.24
**Rural/urban residence of patient**				
Urban (reference)				
Municipal head	1.01	(0.40;	2.54)	0.99
Scattered rural	0.58	(0.27;	1.26)	0.25
**Importance of religion for the caregiver**				
Very important (reference)				
Important	1.24	(0.68;	2.26)	0.55
Not important	3.32	(0.79;	13.91)	0.17
**Caregiver´s relationship with the patient**				
Partner (reference)				
Father/mother/son/daughter	0.96	(0.44;	2.07)	0.93
Brother/sister	0.47	(0.14;	1.59)	0.31
Other	1.53	(0.41;	5.61)	0.59
**Patient age**				
Age 75–93 (reference)				
Age 18**–**44	3.80	(1.33;	10.84)	0.04*
Age 45**–**59	0.77	(0.34;	1.71)	0.58
Age 60**–**74	0.61	(0.30;	1.24)	0.25
**Coliving with the patient**				
No (reference)				
Yes	0.56	(0.30;	1.04)	0.12
**Patient had partner**				
Yes (reference)				
No	1.93	(1.01;	3.67)	0.09*

An OR greater than 1 indicates a higher level of suffering

Sample size for analysis (n = 174), with α = 0.10

The partnership status of the patient was included in the final model, even though it did not behave as a confounder. This decision was made because of the different distribution among responders vs. non-responders. Caregivers of patients who did not have a partner reported a higher level of suffering (OR 1.93 (90%CI; 1.01–3.67)), adjusting for all other variables.

The results of the likelihood ratio test in which the reduced model was compared to the full model with all variables mentioned in the methods section showed no statistically significant differences between the two models (p = 0.85). There were no problems of collinearity between the included variables.

## Discussion

The results of our study showed a high level of suffering in patients who died of cancer in the participating institutions, which was associated with unclear communication with the doctor, treatments inconsistent with the wishes of the patient, palliative care in an outpatient setting, and a younger patient age. The scarce international literature on this topic reports moderate to severe levels of suffering in 27 to 81% of cancer patients attended in secondary care centers [8]. The proportion in our study is higher, perhaps related to the fact that our patients were attended in a tertiary care setting, were all patient at the end of their life. The high proportion of suffering may also be related to the high proportion of hospital deaths in our study: other studies have previously reported an intermediate level of suffering [24] and variations in the level of suffering during the last year of the patient’s life associated with hospitalizations and the use of palliative or hospice care [9].

The high amount of reported suffering may also be related to reports by proxies, potential suboptimal symptom control or suffering from things that palliative care in Colombia is currently not focusing on, such as suffering from the knowledge of imminent death, from worries for those who are left behind, financial worries [25], suffering from having to go to the palliative care services in often suboptimal circumstances. These issues may increase suffering, and may even be a threat to the integrity of the patient, but are cannot be solved completely [26]. Ruijs et al. described similar symptoms of unbearable suffering for patients in the Netherlands, who described among the unbearable aspects impaired activities, feeling dependent, help needed with housekeeping, not being able to do important things, trouble accepting the situation, being bedridden and loss of control [8]. The high proportion of suffering observed is certainly a matter of concern and reason to keep focusing on improving the end-of-life care for cancer patients in Colombia.

Our results may not reflect the reality for Colombia as a whole: patients in our study had a higher probability of receiving palliative care as they had been cared for in institutions that provide these services – which in many parts of the country are absent – therefore the level of suffering in more remote areas may be even higher.

In our study, unclarity in the communication process with the medical professionals was associated with increased suffering. Communication has been recognized as a challenge for health professionals worldwide [18, 27]. A poor communication regarding the prognosis and the preferences of care during the end-of-life between doctor and patient, increases the risk of the use of medical procedures which are inconsistent with the wishes of the patient [3], which is known to cause suffering [28].

The wishes of the patient could include stopping treatments with curative intent at the end of life, or intensifying measures to prolong life [29]. In our study, the application of procedures inconsistent with the patient’s wishes were reported in 15% of the deceased. This relatively high frequency may be due to a lack of communication and knowledge on the part of physicians of the wishes for end-of-life care [10]. Another study investigating the treatment decisions made for the deceased patients of this study [19] reported that only 6% of doctors were aware of the presence of advance directives in their patients [30], which aligns with our findings as reported by caregivers. Unfortunately, we did not have information regarding the content of the patients´ wishes: stopping interventions with curative intent at the end of life, or intensifying measures to prolong life. Given the importance of this information, we suggest including these among the variables to be measured in future studies.

Early and systematic referral to palliative care has shown benefits in increasing its use and reducing aggressive treatments at the end-of-life [31]. Patients who received palliative care have been shown to have a better quality of life [32–34]. Our results showed that most of the patients (n = 155, 88.6%) received palliative care in its different modalities, yet the majority suffered moderately to severely, even a considerable percentage of patients in whom was reported suffering due to life prolonging treatments had received palliative care too. Unfortunately, the information available did not permit distinguishing if they received timely care, nor the frequency or content of the information provided.

Our data showed a higher level of suffering at the end of life of patients who received palliative care in an outpatient versus home-based setting. A plausible explanation for this finding is the lack of continuous schedules in the outpatient clinics (only day-time attention); patients can go to the emergency room to manage their symptoms, but, unfortunately, the perceived quality of care through this route is not always the best [35].

High rates of in-hospital deaths among cancer patient have been linked to increased unnecessary use of chemotherapy and diagnostic imaging before death [36], and thus have been identified as an indicator of poor quality of life at the end of life [17]. Our study had a higher proportion of hospital deaths than the average in the country. The fact that patients were identified at the end-of-life in the three participating hospitals is likely to have created a selection bias—with a higher probability of including patients who would die in the hospital.

It is important to remember that home may not always be the appropriate location for the patient’s death [37]; some hospital deaths may be associated with requirements for optimal symptom control that can only be obtained in a hospital setting. Additional reasons for patients to prefer hospital deaths in Colombia include fear of not knowing how to react to the events that accompany death, the relationship and opportunity with the palliative care specialist, non-acceptance of the end-of-life process [18, 35], and even includes worries regarding difficulties of obtaining the death certificate for home deaths— (personal communication with Dr. Sanchez, clinical oncologist).

The distribution of patient characteristics such as age, sex, and type of cancer were similar to those reported in the literature [36]. In our study, a patient age range between 18 and 44 years was associated with a higher reported level of suffering compared to an age range of 75 to 93 years, probably related to greater psychosocial and spiritual suffering among bereaved caregivers of losing a dear one who is perceived to not have been able to complete their life [38].

## Strengths and limitations

This is the first study from Latin America on factors influencing the suffering of patients who died from cancer. The results of this study may serve as input for studies that evaluate causality, as well as programs that seek to improve end-of-life care.

One of our limitations is that we do not know the state of preparation of the caregiver for the moment of death, whether there was psychological support for the patient and the family, in addition to the multidisciplinary management that could provide support to the cancer patient. We believe that this information, given its relevance, should be considered in future studies.

The mentioned question of the global measurement of suffering does not include the answer option of “no suffering at all”–which may have influenced results. In addition, the time horizon of this question is related to the exact moment of the patient’s death; if other time frames had been used, the perception of the suffering reported by the caregiver may have been different.

Another limitation of these studies is that the level of suffering of the patients during their last week of life was measured through the opinion of proxies (caregivers). It would be helpful to be able to count on the patient as the primary source of information about their own experiences, since the caregiver may report a lower quality of life than would be reported by the patients themselves [39]; this could be influenced by bereavement, the time that caregivers could share with the patient and their perspectives on the experience lived by the deceased patient. It is for this reason that it is advisable to carry out measurements in longitudinal studies that allow knowing the level of agreement between both [39]; Unfortunately, in our study we do not have these measurements.

The study designs favor recall bias: those family members who reported a higher level of suffering may have had a greater tendency to remember the factors associated with suffering compared with the family member who reported a low level of suffering. However, as most participating caregivers were very close relatives of the patient, we feel the information provided is sufficiently reliable.

The global measure of the level of suffering may be underestimated due to a greater representation of patients with a partner in the group of responders whose level of suffering is usually reported as low.

### Clinical implications

Understanding the factors associated with the suffering that patients who die of cancer is very relevant–recent discussions have arisen in Colombia about “death with dignity”—. A dignified death basically consists of “dying in peace”, and avoiding futile suffering, which in turn are the central foundations of a good death.

It is necessary to maintain continuous communication throughout the disease with the patient and his family, the progression of the disease requires that conversations be periodically addressed regarding the prognosis and goals of care, these moments will also allow exploring situations that may be triggering anguish and suffering in the patient and his family. There are interventions to improve this communication [40]; some focused on improving the management of difficult conversations between doctors and their patients [41], others seek to guide the patient in the questions to ask their oncologist during consultations and help improve the agreement between doctors and patients regarding the treatments received at the end of the life [29, 42]. The use of such interventions should be evaluated in Colombia and, if effective, should be integrated in the training of professionals, and in the care of cancer patients.

Work must continue to strengthen the infrastructure of palliative care units in all its modalities, diseases such as cancer that are associated with a high level of suffering at the end of life require comprehensive care. The strategies that could provide a great benefit to the patient and their family include telephone counseling without restrictions on their time availability, in addition to a palliative emergency service that provides a more comfortable environment for the management of medical complications and even an end of life worthy with better accompaniment and privacy.

## Conclusion

Our results show high levels of suffering of cancer patients at the moment of death, which seem to be related to unclear communication with the physician, treatment inconsistent with the patient’s wishes, and having received outpatient palliative care. Diseases such as cancer associated with a high level of suffering at the end-of-life require comprehensive care and a work continuum in the strengthening of the palliative care capabilities in Colombia.

## Electronic supplementary material

Below is the link to the electronic supplementary material.

Supplementary Material 1

Supplementary Material 2

Supplementary Material 3

## Data Availability

Deidentified participant data, protocols, statistical analysis plans are available upon reasonable request to Esther de Vries, ORCID identifier 0000-0002-5560-2258.

## Abbreviations

BDUA: Base de Datos Única de Afiliados
HUSI: Hospital Universitario San Ignacio Bogotá
HUSJ: Hospital Universitario San José Popayán
IC: Confidence Interval
INC: Instituto Nacional de Cancerología Bogotá
IQR: Interquartile Range
OR: Odds Ratio
