# Supplementary material for: Factors associated with suffering from dying in patients with cancer: a cross-sectional analytical study among bereaved caregivers
Source: BMC Palliat Care. 2023 Apr 21;22:48. doi: 10.1186/s12904-023-01148-x (PMC10120203; doi:10.1186/s12904-023-01148-x)
Supplement: Supplementary file 1 — Supplementary Material 1 [file 12904_2023_1148_MOESM1_ESM.docx]

**Supplementary table 1. Study variables**

| **Name** | **Measurement level** | **Unit** | **Source** |
| --- | --- | --- | --- |
| Patient age | Quantitative of reason | -Years old | Patient’s clinical history |
| Sex | Nominal qualitative | -Male  -Female | Patient’s clinical history |
| Patient had partner | Nominal qualitative | -With partner  -Single | Question 6 caregiver survey |
| Health affiliation scheme* | Nominal qualitative | -Contributory  -Subsidized | Patient’s clinical history |
| Place where the patient died | Nominal qualitative | -Home (house/apartment, someone close’s house/apartment  -Hospital | Question 14 caregiver survey |
| Educational level reached by the patient | Qualitative ordinal | - No formal education  -Basic (primary, high school, technical education)  -University/higher education (graduate, university, postgraduate/specialization, postgraduate/master’s degree, postgraduate/doctorate) -Don’t know | Question 13 caregiver survey |
| Center of attention | Nominal qualitative | -HUSI  -HUSJ  -INC | Caregiver survey.  Case identifier code |
| Days between the interview and the death of the patient | Quantitative of reason | -Days | Question 10 caregiver survey  It was constructed by subtracting the date of the interview from the date of the patient's death. |
| Importance of religion for the caregiver | Qualitative ordinal | -Very important  -Important  -Not important | Question 20 caregiver survey |
| Level of involvement of the interviewee in patient care | Qualitative ordinal | -Very involved (primary caregiver)  -Somewhat involved (accompanying in some processes/little involved) | Question 21 caregiver survey |
| Procedures inconsistent with patient wishes | Nominal qualitative | -Yes  -No | Question 32 caregiver survey |
| Palliative care modality | Nominal qualitative | -Domiciliary  -Ambulatory -Hospitalized -Did not receive | Question 16 caregiver survey |
| Living condition | Qualitative  nominal | -Others, please specify  -Lives alone  -Lives with partner  -Lives with partner and children -Lives with children --Lives with parents | Question 8 caregiver survey |
| Treatments to prolong of life | Qualitative  nominal | -Yes  -No | Question 26 caregiver survey  CEQUEL** |
| Prolongation of life during the dying process | Qualitative  nominal | -Yes  -No | Question 27 caregiver survey  CEQUEL |
| Greater suffering due to prolongation of life | Qualitative  nominal | -Yes  -No | Question 28 caregiver survey  CEQUEL |
| Type of cancer diagnosed | Nominal qualitative | -Solid  -Hematological | Question 3 caregiver survey |
| Rural/urban residence of patient | Nominal qualitative | -Urban  -Municipal head  -Scattered rural | Question 9 caregiver survey |
| Offspring | Qualitative  nominal | -Yes  -No | Question 11 caregiver survey |
| Children’s age | Qualitative  ordinal | - Does not have children  - Children aged ≥18 (over 30 years old/ between 18 and 30 years old)  - Children <18 (between 12 and 17 years old/under 12 years old)  -Does not know | Question 12 caregiver survey |
| Clarity in the information received by the doctor | Nominal qualitative | -Yes  -No | Question 29 caregiver survey  CEQUEL |
| Caregiver´s relationship with the patient | Qualitative  nominal | -Other  -Partner  -Father/mother /son/daughter/  -Brother/sister | Question 5 caregiver survey |
| Coliving with the patient | Qualitative  nominal | -Yes  -Not | Question 7 caregiver survey |
| Sex of caregiver | Qualitative  nominal | -Female  -Male | Question 81 caregiver survey |
| Presence of someone during the death of the patient | Qualitative  nominal | -Yes  -No, doesn't know | Question 69 caregiver survey  QODD*** |
| Level of suffering | Qualitative ordinal | -(1 to 2) Minimally  -(3 to 5) Moderately  -(6 to 7) Extremely | Question 39 caregiver survey |

*The Colombian health system provides universal coverage to 99.4% of its population through insurance two large schemes: the subsidized scheme (47.2%), to which people without payment capacity belong, and the contributory scheme (48.5 %), which is financed by contributions from the labor force (employees and employers), there is also an exception regime (4.2%) to which the public force and some institutions^11,12^ belong.

** (CEQUEL) Caregivers Evaluation of Quality of End of Life Care

***(QODD) Quality of Dying and Death Questionnaire

**Supplementary table 2. Proportional odds test**

| **Variable** | | **b[polr]** | **b[>minimally]** | **b[>moderately]** | **x2** | **gl** | **Pr(>chisq)** |
| --- | --- | --- | --- | --- | --- | --- | --- |
| **Total** | |  |  |  | 17.53 | 22 | 0.73 |
| **Place where died** |  | |  |  |  |  |  |
| Home (reference) |  | |  |  |  |  |  |
| Hospital | | 0.171 | 0.298 | 0.303 | 0 | one | 0.99 |
| **Clarity in the information received by the doctor** | |  |  |  |  |  |  |
| Yes (reference) | |  |  |  |  |  |  |
| No | | 0.814 | 0.504 | 0.94 | 0.77 | one | 0.38 |
| **Palliative care modality** | |  |  |  |  |  |  |
| Domiciliary(reference) | |  |  |  |  |  |  |
| Ambulatory | | 1.12 | 1.86 | 0.743 | 1.74 | one | 0.19 |
| Hospitalized | | 0.352 | 0.601 | 0.163 | 0.26 | one | 0.61 |
| Did not receive | | 0.448 | 0.169 | 0.373 | 0.04 | one | 0.83 |
| **Procedures inconsistent with the wishes** | |  |  |  |  |  |  |
| No (reference) | |  |  |  |  |  |  |
| Yes | | 1.07 | 1.51 | 0.976 | 0.4 | one | 0.52 |
| **Treatments to prolong of life** | |  |  |  |  |  |  |
| No (reference) | |  |  |  |  |  |  |
| Yes | | -0.0161 | -0.473 | 0.205 | 1.51 | one | 0.22 |
| **Prolongation of life during the dying process** | |  |  |  |  |  |  |
| No (reference) | |  |  |  |  |  |  |
| Yes | | 0.361 | 0.342 | 0.361 | 0 | one | 0.97 |
| **Greater suffering due to prolongation of life** | |  |  |  |  |  |  |
| No (reference) | |  |  |  |  |  |  |
| Yes | | 0.286 | 0.501 | -0.0298 | 0.68 | one | 0.41 |
| **Type of cancer** | |  |  |  |  |  |  |
| Solid (reference) | |  |  |  |  |  |  |
| Hematological | | -0.658 | -0.526 | -0.793 | 0.11 | one | 0.74 |
| **Rural/urban residence of patient** | |  |  |  |  |  |  |
| Urban (reference) | |  |  |  |  |  |  |
| Municipal head | | 0.0101 | 0.432 | -0.165 | 0.44 | one | 0.51 |
| Scattered rural | | -0.545 | -0.647 | -0.654 | 0 | one | 0.99 |
| **Importance of religion for the caregiver** | |  |  |  |  |  |  |
| Very important (reference) | |  |  |  |  |  |  |
| Important | | 0.217 | -0.125 | 0.273 | 0.65 | one | 0.42 |
| Not important | | 1.2 | 15.6 | 1.09 | 0 | one | 0.99 |
| **Caregiver´s relationship with the patient** | |  |  |  |  |  |  |
| Partner (reference) | |  |  |  |  |  |  |
| Father/mother/son | | 0.423 | 0.392 | 0.177 | 0.04 | one | 0.83 |
| Brother/sister | | -0.0435 | 0.0627 | -0.217 | 0.18 | one | 0.67 |
| Other | | -0.758 | -1.23 | -0.784 | 0.21 | one | 0.65 |
| **Patients age** | |  |  |  |  |  |  |
| Age 75–93 (reference) | |  |  |  |  |  |  |
| Age 18**–**44 | | 1.34 | 1.77 | 1.39 | 0.14 | one | 0.71 |
| Age 45**–**59 | | -0.266 | -0.31 | -0.162 | 0.05 | one | 0.82 |
| Age 60**–**74 | | -0.496 | -0.575 | -0.491 | 0.02 | one | 0.89 |
| **Coliving with the patient** | |  |  |  |  |  |  |
| No (reference) | |  |  |  |  |  |  |
| Yes | | -0.576 | -0.504 | -0.647 | 0.09 | one | 0.77 |
| **Patient had a partner** | |  |  |  |  |  |  |
| Yes (reference) | |  |  |  |  |  |  |
| No | | 0.66 | 0.316 | 0.928 | 1.45 | one | 0.23 |
