# Supplementary material for: Factors associated with suffering from dying in patients with cancer: a cross-sectional analytical study among bereaved caregivers
Source: BMC Palliat Care. 2023 Apr 21;22:48. doi: 10.1186/s12904-023-01148-x (PMC10120203; doi:10.1186/s12904-023-01148-x)
Supplement: Supplementary file 2 — Supplementary Material 2 [file 12904_2023_1148_MOESM2_ESM.pdf]

Survey for relatives on the end of life of an oncology patient

Demographic questions

Instructions for interviewer:

The interviewer should insert personal information in the questions, when appropriate. For example, use the patient's name where the word [PATIENT] appears.

As I had previously explained to you, we are conducting a study to learn about the end of life of cancer patients. For this, we are contacting relatives or caregivers of patients who have died from cancer. In this interview, we would be asking you to provide information about [PATIENT NAME]. How would you like us to refer to him / her?

This survey consists of several parts, some about the family situation of [PATIENT], some about yourself, others about health and medical care in the last week of [PATIENT] life and finally some about the impact that the disease of [PATIENT] had in his family.

Each question will have some response categories, I will be informing you.

It is important that you know that your participation is completely voluntary, and that it will be recorded. This recording will only be used by me to be able to complete some questions if necessary. Once that verification is complete, I will be deleting the recording. Your personal data or those of [PATIENT] will be recorded for the study.

You can decide to stop participating in the interview at any time, without any consequences for you.

Do you have any questions?

Well ... we start with some questions about the situation of [PATIENT]

1. Informed consent mode

☐ Verbal (recorded)

☐ Electronic -

☐ emailElectronic -

☐ Verbal form (without recording)

2. Case number (equal to Study A - physicians)

3. What type of cancer had [PATIENT] been diagnosed with?

- Breast Ca
- Prostate Ca
- Stomach Ca
- cervical cancer
- Lung cancer
- Colon and Rectum
- Don't know

Other (specify)

4. How long ago did you find out about [PATIENT] 's diagnosis?

5. How was he related to [PATIENT]?

- Couple
- Son/daughter
- Father mother
- Brother / sister

other, please specify:

6. ¿Did [PATIENT] have a partner?

If the couple is the interviewee - do not ask but fill in "yes"

- Yes
- Not
- Does not Know

7. [PATIENT lived with you?

- Yes
- Not

8. Who (else) did [PATIENT] live with?

Use the ¨mas¨ when the respondent answered yes to the previous question

- Alone, independent
- Without a partner with children
- With couple
- With parents
- With partner and children
- Does not know
- Other (s), please specify

[ ]

9. Did [PATIENT] live in urban, rural, municipal or dispersed rural areas?

- Urban
- Municipal head
- Scattered rural
- other (specify)

[ ]

10. How long ago did [PATIENT] die? For the purposes of the study it would be important to have the exact date, if you know it.

In case of not having exact date, use approximate date or option "don't know"

Know exact date Day / Month / Year

Date

DD / MM / YYYY

Know approximate date

Date

DD / MM / YYYY

Don't know, place 01/01/1990

Date

DD / MM / YYYY

11. Did [PATIENT] have children?

- Not
- Dont Know
- Yes, please indicate the number ofchildren:

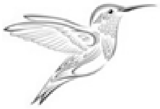

Survey for relatives on the end of life of an oncology patient

12. How old were your children when [PATIENT] died)? Please specify ages of all children.

- [ ] Older than 30 years
- [ ] Between 18 and 30
- [ ] Between 12 and 17 years old
- [ ] Under 12 years old
- [ ] Does not know

13. What educational level did [PATIENT] reach?

- Primary
- high school
- Technical education
- Graduate
- college
- other, please specify:
- Postgraduate – specialization
- Postgraduate - Master's
- Postgraduate - doctorate
- Don't know

14. Did [PATIENT] die at home or in the hospital?

- In your house / apartment
- In someone's house / apartment close to you
- In hospital or clinic
- Other, please specify:

15. Did [PATIENT] receive palliative care?

- Yes
- Not
- I'm not sure

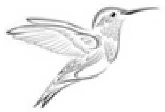

Survey for relatives on the end of life of an oncology patient

16. The hospice care was mainly home, outpatient or hospitalized?

- Domiciliary
- Ambulatory
- Hospitalized
- Other, please specify

17. Was [PATIENT] treated for pain, nausea and vomiting, shortness of breath, and other symptoms at the end of life?

- Yes
- Not
- I'm not sure
- Comments

Survey for relatives on the end of life of an oncology patient

P2: family member's information

Now, we have a few questions about yourself.

18. Do you practice any religion? If so, which one?

Although the word "religion" is used, they can also be themes of "spirituality".

- Not
- Yes catholic
- Yes christian
- Yes jew
- Other, please specify
- Yes muslim
- Yes hindu
- Yes, buddhism
- Not a specific religion,I do believe in a "God" or I am spiritual

Survey for relatives on the end of life of an oncology patient

19. How often do you attend religious services or meetings of your religious community?

Although the word "religious" is used, they can also be themes of "spirituality".

- Never
- A few times a year
- More or less monthly
- More than once a month
- Weekly
- More than once a week

20. How important is religion to you?

If indicated to be "spiritual" change to "spirituality"

- Very important
- Important
- Not important

21. How involved were you in the care of [patient] in the last few weeks prior to his death?

- ◯ He was very involved, one of the main caretakers
- ◯ Somewhat involved, accompanying in some processes
- ◯ Little involved
- ◯ Uninvolved
- ◯ other (specify)

22. Were you present at the time of [PATIENT] death?

- ◯ Yes I was present
- ◯ No I was not present

23. In hindsight, would you have wanted to be present at the moment of death?

- ◯ Yes
- ◯ Not
- ◯ I do not care
- ◯ If not, why not?

24. Was there some kind of parting with [PATIENT] before his death?

- ◯ Yes
- ◯ Not

If yes, please summarize farewell

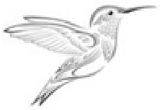

Survey for relatives on the end of life of an oncology patient

Caregivers Evaluation of Quality of End of Life Care (CEQUEL)

Instructions for interviewer:

The interviewer should insert personal information in the questions, when deemed appropriate. For example, use the patient's name where the word [PATIENT] appears.

This instrument is designed to assess the quality of life of the patient just before death, in the hospital.
- If the patient was hospitalized at the time of death, please use the phrase: "During this last week…. "
- In the scenario in which the patient was not hospitalized at the time of death or in the month prior to death, ask about "the care given by__________________________(and determines who was the caregiver) during the last week of the patient's life.

Now we move on to a part of the interview where there will be questions about [PATIENT] 's last week of life.

25. I am going to ask you some questions about the last week of [PATIENT] life.
Where was [PATIENT] during the last week of life?

- ◯ Hospital or home care
- ◯ At home or home withouthealth care
- ◯ other (specify)

[ ]

26. During this past week, was [PATIENT] 's life prolonged by medical interventions longer than you would have expected?

Let the interviewee answer the question, do not give "I don't know" as an option so that the wild card does not become. Obviously, if you really don't know, use this option.

- ◯ Yes
- ◯ Not
- ◯ Dont Know
- ◯ comments

[ ]

27. During this past week, was [PATIENT] 's life being prolonged by medical interventions when he / she was, as far as you know, already in the process of dying?

- Yes
- Not
- Dont Know
- comments

28. During this last week, was [PATIENT] 's life prolonged by the medical treatments he received and did this cause him greater suffering?

- Yes
- Not
- Dont Know

29. During this last week, was the information that the doctor gave you about the treatment and the process before death clear?

- Yes
- Not
- Dont Know
- comments

30. During this past week, did the treating physician or someone from the medical team who treated [PATIENT] talk with him / her about his / her preferences in terms of medical treatment?

- Yes
- Not
- I dont know

31. Did [PATIENT] express any wishes about medical treatment at the end of life?
They can be formal or informal advance directives or verbal expressions about your wishes.

- I did have an advance directive
- Yes, informally
- No, he did not express his wishes
- I dont know
- Other (specify) / space for comments

32. During this last week, was there any medical procedure or treatment that was applied to him / her that was inconsistent with his / her previously stated wishes or that he / she would not have wanted?

- Yes
- Not
- I dont know
- If so, please comment on the treatment or the unwanted situation

33. During this past week, did the doctors you talk to about treating [PATIENT] listen to your concerns about it?

- Yes
- Not
- I dont know

34. During this past week, did you or your family receive any information about what to expect while [PATIENT] was passing away?

- Yes
- Not
- I dont know

35. Would you have appreciated receiving any additional information about what to expect while [PATIENT] was passing away?

- Yes
- Not
- other (specify)

36. Did you or your family ever receive information about medications that would be used to manage pain, shortness of breath, or other symptoms from [PATIENT]?

- Yes
- Not
- I dont know
- Comments

37. Would you have liked (more) information about medications?

- Yes
- Not

38. During this last week, how often were you or your family members informed about the situation / condition of [PATIENT]?

- Forever
- Frequently
- Sometimes
- Never
- I don't know / other: specify

Survey for relatives on the end of life of an oncology patient

Nature of Death

Nature of the Death (NAT)

In the next section of the survey, we are going to ask a few things related to the time of death of [PATIENT]. The answers are on a scale of 1 to 7 points. In each question I will be informing you about the score on the scale. For each question, please mention the number that best describes how you feel.

39. From 1 to 7, how much do you think [PATIENT] suffered when he died?

	Minimally			Moderately			Extremely
Suffering	○	○	○	○	○	○	○

other (specify)

40. From 1 to 7, in your opinion, how calm or traumatic did [PATIENT] death seem to you?

	Peaceful			Moderate			Traumatic
Death	○	○	○	○	○	○	○

other (specify)

41. From 1 to 7, how much did [PATIENT] suffer compared to what you would have expected?

	A lot less			Same			A lot ofmore
Suffering	○	○	○	○	○	○	○

other (specify)

Survey for relatives on the end of life of an oncology patient

Quality of Death and Dying (QODD)

I am going to ask you some aspects of the last week of [PATIENT] life. Now the scale changes a bit: the questions have answers on a scale from "never" to "all the time", with "never" being one and "all the time" being 5 points on the scale. There are no right or wrong answers, the important thing is that you provide us with your impression of the situation of [PATIENT] in his last week of life.

42. From 1 to 5, how often did [PATIENT] seem to have his pain controlled?

	Never		Enoughtimes		All the time
Pain control	○	○	○	○	○

other (specify)

43. From 1 to 5, how often did [PATIENT] have control over what was happening in their environment?

	Never		Enoughtimes		All the time
Control over what was happening in your environment	○	○	○	○	○

other (specify)

44. From 1 to 5, how often was [PATIENT] able to feed himself?

	Never		Enoughtimes		All the time
Feeding alone	○	○	○	○	○

other (specify)

45. From 1 to 5, how often did [PATIENT] have control of his sphincters, both urine and bowel movements?

	Never		Enoughtimes		All the time
Toilet training	○	○	○	○	○

other (specify)

46. From 1 to 5, how often was [PATIENT] able to breathe without much difficulty?

	Never		Enoughtimes		All the time
Difficulty breathing	○	○	○	○	○

other (specify)

47. From 1 to 5, how often did [PATIENT] seem uneasy about death?

	Never		Enoughtimes		All the time
Peace of mind in the face of death	○	○	○	○	○

other (specify)

48. From 1 to 5, how often did [PATIENT] seem not afraid of dying?

	Never		Enoughtimes		All the time
Not be afraid of dying	○	○	○	○	○

Don't know or other (specify)

49. From 1 to 5, how often did [PATIENT] smile or laugh in those last few days?

	Never		Enoughtimes		All the time
Laughed and smiled	○	○	○	○	○

other (specify)

50. From 1 to 5, how often did [PATIENT] have the energy to do what they wanted?

	Never		Enoughtimes		All the time
Energy to dowhat i wanted	○	○	○	○	○

other (specify)

51. From 1 to 5, how often did [PATIENT] seem to be concerned about overloading their loved ones or becoming a nuisance to them?

	Never		Enoughtimes		All the time
Burden for your loved onesdear	○	○	○	○	○

other (specify)

52. From 1 to 5, how often did [PATIENT] seem to maintain his dignity and respect?

	Never		Enoughtimes		All the time
Dignity and respect	○	○	○	○	○

other (specify)

53. From 1 to 5, how often did [PATIENT] spend time with a spouse or partner (or did not have a partner)?

	Never		Enoughtimes		All the time
Time with spouse or partner	○	○	○	○	○

I did NOT have a partner or other (specify)

54. From 1 to 5, how often was [PATIENT] with your children? (or had no children)

	Never		Enoughtimes		All the time
Frequency withchildren	○	○	○	○	○

I had no children or another(specify)

55. From 1 to 5, how often did [PATIENT] spend time with friends and other family members?

	Never		Enoughtimes		All the time
Time with friends and other family	○	○	○	○	○

other (specify)

56. From 1 to 5, how often did [PATIENT] spend time alone?

	Never		Enoughtimes		All the time
Time alone	○	○	○	○	○

other (specify)

57. [PATIENT] had pets? If yes, how often did [PATIENT] spend time with pets (or had no pets)?

	Never		Enoughtimes		All the time
Time with pets	◯	◯	◯	◯	◯

I had no pets or other (specify)

58. Was [PATIENT] able to find meaning and meaning in his life?

- Yes
- Not
- I dont know

59. Was [PATIENT] accompanied and hugged by those he loved?

- Yes
- Not
- I dont know

60. Was [PATIENT] able to attend important events (graduations, weddings, birthdays)?

- Yes
- Not
- I dont know

Comments observations:

61. Did [PATIENT] have all health costs covered?

- Yes
- Not
- I dont know

62. Was [PATIENT] able to say goodbye to loved ones?

- Yes to everyone
- Yes, to the closest but not to all
- Yes to some
- Not
- I dont know

63. Did [PATIENT] have one or more visits from a religious or spiritual advisor?

- Yes
- Not
- I dont know

64. Did [PATIENT] have a spiritual rite or ceremony before passing away?

- Yes
- Not
- I dont know

65. Did [PATIENT] use a mechanical ventilator (or breathing machine) or dialysis to prolong his life?

- Yes
- Not
- I dont know

66. Was [PATIENT] able to heal bad feelings?

- Yes
- Not
- I dont know

67. Did [PATIENT] have the funeral arrangements in order before passing away?

- Yes, costs were covered (insurance)
- Yes, he had expressed how he wanted thefuneral arrangements
- Yes, both costs andthe arrangements were in order
- Not
- I dont know

68. Was [PATIENT] able to discuss his wishes regarding the care of the dying process with his doctors and others?

- Yes
- Not
- I dont know

69. Was anyone present at the time of [PATIENT] death?

- Yes
- Not
- I dont know

70. At the time of death, [PATIENT] was ...

- Awake
- Sleeping
- In a coma /Inconscious
- Does not know
- Does not respond

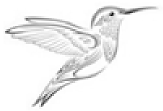

Survey for relatives on the end of life of an oncology patient

COVINSKY FAMILY IMPACT SURVEY

I would like to ask you about some aspects in which [PATIENT] 's illness may have affected other members of the family.

71. Has anyone in your family gotten sick or had stress or tension problems associated with [PATIENT] illness and death that caused you to stop doing your daily activities?

- Yes
- Not
- I do not know
- Comments

72. Did [PATIENT] illness mean having to use all or most of the family's savings?

- Yes
- Not
- I do not know
- Comments

73. Did [PATIENT] 's illness mean the loss of the main source of income for your family?

- Yes
- Not
- I do not know
- Comments

74. Did the costs associated with care for [PATIENT] disease cause the family to move to a less expensive place to live?

- Yes
- Not
- Comments

75. Did the costs associated with care for [PATIENT] necessitate postponing important medical care for someone else in the family?

- Yes
- Not
- I do not know
- Comments

76. Did the costs associated with care for [PATIENT] require postponing education plans or significantly changing plans for any other family member?

- Yes
- Not
- Dont Know
- Comments

77. Please tell me more about these changes (skip if not applicable):

Interview closing

CLOSING QUESTIONS

We would like to know how you felt participating in thisinterview

78. Did you find participating in this interview disturbing or difficult?

- ◯ Not at all or verylittle
- ◯ Something disturbing
- ◯ Moderately disturbing
- ◯ Quite disturbing Very
- ◯ disturbing

79. To what extent do you feel this interview is beneficial / useful for you?

- Very beneficial
- Moderately beneficial
- Little beneficial
- It had no benefit

80. Do you have any other comment about your participation in this interview?

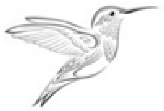

What is the gender of the person who answered this survey?

Survey for relatives on the end of life of an oncology patient

Questions for interviewer

- Male
- Feminine

81. What was the general attitude of the interviewee during the questions?

- Friendly
- Cooperative but not very friendly
- Indifferent
- Suspicious
- Hostile and uncooperative

82. What was the level of general emotional affectation of the interviewee?

- Minimal or no affectation
- Moderately affected
- Affected
- Very affected

83. Please rate the confidence of the interviewee in providing information.

- Highly reliable
- Pretty reliable
- Trustworthy
- UnreliableVery
- unreliable

84. Was the entire interview completed?

- Yes
- Not

Survey for relatives on the end of life of an oncology patient

85. If it was not completed in full, please indicate why:

- Fatigue
- Emotional affectation
- Time extension
- Other, please specify:

86. Space for comments or notes from the interviewer about this interview
