# Supplementary material for: Factors associated with suffering from dying in patients with cancer: a cross-sectional analytical study among bereaved caregivers
Source: BMC Palliat Care. 2023 Apr 21;22:48. doi: 10.1186/s12904-023-01148-x (PMC10120203; doi:10.1186/s12904-023-01148-x)
Supplement: Supplementary file 3 — Supplementary Material 3 [file 12904_2023_1148_MOESM3_ESM.docx]

**Key Messages**

*•* ¿ What was already known?

According to Cassell, suffering manifests itself when the human being experiences an imminent attack on his integrity.

Palliative care reduces the suffering of diseases such as cancer, by providing comprehensive support to the patient and his family.

*•* ¿ What are the new findings?

The communication of the health personnel towards the patient and his family must be continuous and according to the state of the disease.

Clear communication with the patient could reduce the use of treatment inconsistent with the patient’s wishes and, thus, suffering.

Studies are required to evaluate possible barriers in outpatient palliative care.

*•* Implications for practice, theory or policy

Knowing the factors that are associated with greater suffering at the end of life will allow the creation of strategies to improve care for cancer patients and their families.

Results obtained with our study may serve as a guide for studies that assess causality.
